# SAW-Based Hydrogen Sensing: Mechanisms, Design Strategies, and Future Prospects

**DOI:** 10.3390/mi16111227

**Published:** 2025-10-28

**Authors:** Shengzhuo Chen, Jin Chai, Libo Gao, Rongjie Wang, Zhonggang Zhang, Ziming Ren, Hongyan Xu, Yihui Lan, Kezhen Ma, Meng Li

**Affiliations:** 1 School of Marine Engineering, Jimei University, Xiamen 361000, China; 13835918541@163.com (S.C.);; 2 Zhongbei Rongchuang (Xiamen) Perception Technology Research Institute, Xiamen 361000, China; 3 School of Instrument and Electronics, North University of China, Taiyuan 030051, China; 4 Pen-Tung Sah Institute of Micro-Nano Science and Technology, Xiamen University, Xiamen 361102, China; 5 Laoshan Laboratory, Qingdao 266237, China

**Keywords:** surface acoustic wave, hydrogen sensor, response mechanism, hydrogen-sensitive film, signal processing

## Abstract

Hydrogen is widely considered a clean and sustainable energy carrier due to its high energy density, abundant reserves, and zero carbon emissions during use. To ensure safety in hydrogen production, transportation, and utilization, the development of high-performance hydrogen sensors is of great importance. Among various sensing technologies, surface acoustic wave (SAW) sensors have attracted considerable attention due to their unique advantages, including rapid response and high sensitivity, which originate from the excitation and reception of acoustic waves by interdigital transducers and the strong surface disturbance sensitivity of piezoelectric substrates. This paper systematically discusses the sensing mechanisms of SAW hydrogen sensors, analyzes the effects of piezoelectric substrates and hydrogen-sensitive materials on sensing performance, reviews recent progress in hydrogen-sensitive films, and explores optimization strategies in electrode structure design and signal processing. Finally, the main challenges are summarized, and future development directions are outlined, aiming to provide theoretical support for the design and application of high-performance SAW hydrogen sensors.

## 1. Introduction

In modern society, Hydrogen (H_2_) is recognized as a promising clean energy and an important industrial raw material, with its strategic significance continually increasing [1,2,3,4]. However, its inherent physical and chemical properties also pose significant safety risks. Hydrogen has an extremely low density (0.0899 kg/m^3^) and a high diffusion rate, enabling it to disperse rapidly in the event of a leak. Additionally, it exhibits a very low ignition energy over a broad concentration range (4–75%). Because H_2_ molecules are small, colorless, and odorless, leaks are difficult for humans to detect promptly. Consequently, H_2_ can easily leak during storage, transportation, and use, leading to serious safety hazards. Therefore, rapid and accurate detection of hydrogen concentration in the environment is critically important.

To meet this demand, researchers have gradually introduced surface acoustic wave (SAW) technology into the field of hydrogen detection. SAW is a kind of coupled elastic wave propagating along the surface of solid medium, which was firstly discovered and described by Rayleigh in 1885 [5], Gibson, D. S further elaborated the propagation characteristics of SAW on solid surfaces in 1962, which laid the theoretical foundation of the subsequent applied research of SAW technology, and Voltmer landmark research demonstrated that by arranging fork finger electrodes on the surface of piezoelectric materials and applying RF signals, surface acoustic waves can be efficiently excited and received, and this discovery marked the beginning of SAW device technology [6]. Since then, thanks to the advances in semiconductor integrated circuit processes, SAW devices have been miniaturized while their commercial value has become increasingly prominent. Due to their excellent frequency selectivity and signal processing capabilities, SAW devices have been widely used in the fields of wireless communications, aerospace and medical technology [7,8,9].

## 2. Overview of Hydrogen-Sensing Technologies

In recent years, with the layout of the hydrogen energy industry and the rapid development of the industrial revolution, the market demand for high-sensitivity and fast-response hydrogen sensors has increased. According to ISO 26142 [10], published by the International Organization for Standardization (ISO), hydrogen sensors must comply with the performance requirements listed in Table 1. Current research on hydrogen sensors encompasses thermal conductivity, optical, electrochemical types, resistive, field effect transistor (FET) and acoustic. However, thermal conductivity hydrogen sensors detect hydrogen concentration by measuring the change in resistance of a thermal-sensitive element, which occurs as hydrogen alters the thermal conductivity of the surrounding gas and thereby removes heat from the element. These sensors offer a wide detection range, simple structure, and good long-term stability. However, they are susceptible to interference from other gases and generally exhibit relatively high power consumption [11,12,13]. Optical hydrogen sensors typically consist of a light source, a detector, and an optical system. Their operating principle is based on hydrogen adsorption, which induces changes in the electronic structure of the optically sensitive material, leading to measurable variations in reflectance, transmittance, or refractive index that can be detected by the sensor. Because the sensing signal is optical, these sensors offer advantages such as low baseline drift, high sensitivity, low detection limits, and inherent safety. However, they are often characterized by complex structures, high packaging costs, and limited long-term stability [14,15,16,17]. Electrochemical hydrogen sensors typically employ a three-electrode configuration, consisting of a working electrode, counter electrode, and reference electrode, in conjunction with an electrolyte and a diffusion membrane. Their operating principle relies on the electrochemical oxidation of hydrogen at the working electrode surface, generating a current signal proportional to the hydrogen concentration, thereby enabling quantitative detection. This type of sensor exhibits high selectivity, low detection limit, and fast response; however, the electrolyte is prone to corrosion or evaporation, which shortens the overall device lifetime [18,19,20,21]. The working principle of resistive hydrogen sensors lies in the fact that the physical adsorption or chemical reaction between hydrogen molecules and the sensing material surface alters the carrier concentration or mobility, thereby inducing a measurable change in resistance. This type of sensor exhibits fast response and high sensitivity [22,23]; However, it suffers from poor selectivity, and most devices require elevated operating temperatures, resulting in relatively high power consumption [24]. The operating principle of Field-Effect Transistor (FET) hydrogen sensors lies in the interaction between hydrogen and the metal gate or catalytic layer, which alters the surface potential barrier of the semiconductor channel, thereby modulating the channel current for hydrogen detection. This type of sensor features high sensitivity, a low detection limit [25], and easy integration with CMOS circuits. However, its performance is susceptible to environmental factors [26].

**Figure 1:**
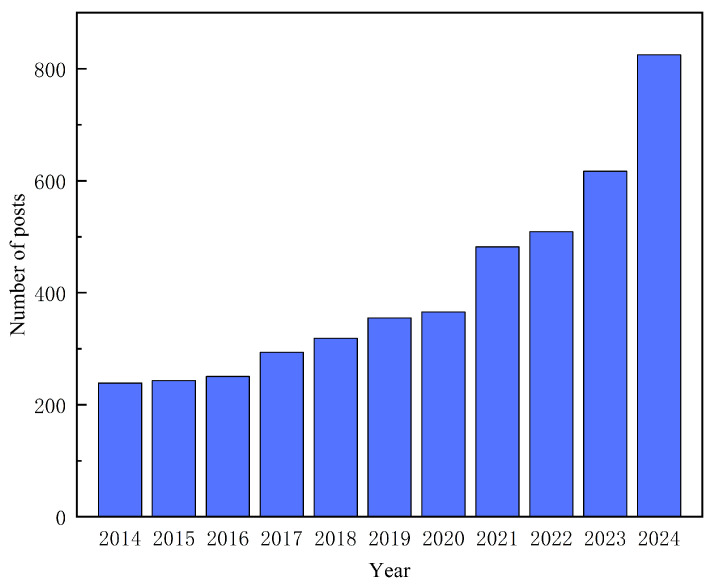
Annual publication volume of SAW hydrogen sensors in the past decade (data source: Elsevier, search keyword: SAW hydrogen sensor).

**Figure 2:**
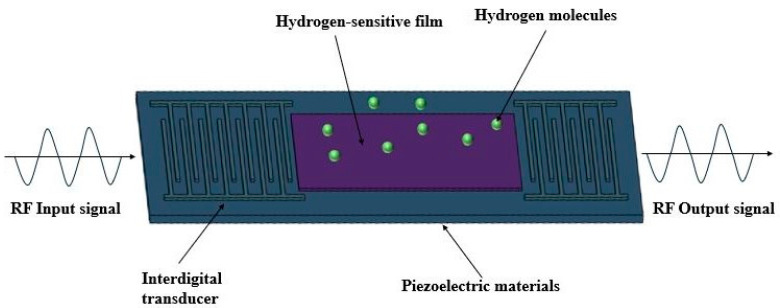
Schematic diagram of the working principle of the SAW hydrogen sensor.

In contrast, SAW devices demonstrate remarkable advantages. Through optimization according to specific standards, SAW devices can achieve highly precise and reliable sensing performance. As illustrated in Figure 1, SAW hydrogen sensors have attracted significant research interest over the past decade. In terms of sensitivity, SAW devices are inherently highly responsive to changes in surface loading due to their surface wave propagation characteristics, providing significant sensing potential. Regarding selectivity, SAW devices achieve gas-specific responses by depositing functionalized sensing films along the acoustic propagation path. These films can be tailored to hydrogen properties, thereby enhancing hydrogen adsorption while minimizing interference from other gases, enabling highly selective detection of hydrogen [27,28,29]. The rapid response of SAW devices is mainly attributed to the confinement of acoustic energy near the surface of the piezoelectric substrate. Upon hydrogen adsorption, slight changes occur in the film’s mass, conductivity, and viscoelasticity, which are immediately translated into frequency or phase shifts, allowing fast detection of hydrogen concentration. Moreover, SAW devices exhibit strong structural stability, ensuring long-term operational reliability even under harsh environmental conditions. As shown in Figure 2, the SAW hydrogen sensor structure consists of fork finger electrodes, a piezoelectric substrate, and a hydrogen-sensitive thin film. The acoustic signal is excited by the fork finger electrodes on one side through the acoustic wave transmission area and received by the fork finger electrodes on the other side. With its unique advantages of simple design, passivity, high sensitivity, fast response time and ease of integration [30,31,32,33,34], SAW technology has become a research hotspot in gas sensing, particularly for hydrogen concentration detection, where safety and accuracy requirements are extremely high [35,36].

In addition to their excellent performance in traditional RF communications, the inherent surface wave propagation characteristics of SAW devices make them extremely sensitive to changes in surface loading, giving them great sensing potential. This sensitivity allows SAW devices to detect gas concentrations in real time by coating the propagation path with a specific hydrogen-sensitive film. H_2_ adsorption can cause small changes in the film’s mass, conductivity, or viscoelasticity, resulting in measurable shifts in the frequency and amplitude of the SAW.

## 3. Response Mechanism of SAW Hydrogen Sensors

**Figure 3:**
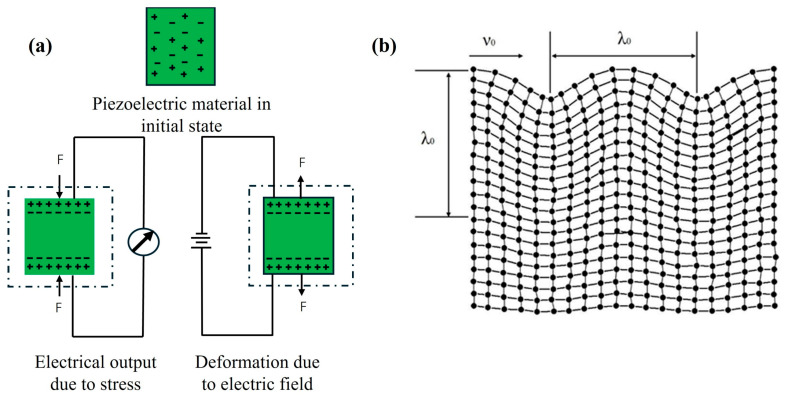
(**a**) Schematic diagram of the piezoelectric effect. (**b**) Rayleigh wave particle trajectory diagram.

As shown in Figure 3a, the working principle of the surface acoustic wave is based on the piezoelectric effect of piezoelectric materials. When an external force is applied, the internal charges are redistributed and generate an electric field. Conversely, when an electric field is applied to the surface of the piezoelectric substrate, it induces mechanical strain within the material, thereby exciting the surface acoustic wave. This bidirectional conversion between mechanical and electrical energy is referred to as the electromechanical coupling effect. As shown in Figure 3b, the acoustic wave energy is primarily concentrated within one or two wavelengths from the substrate surface, and its propagation characteristics are highly sensitive to surface perturbations. When hydrogen is adsorbed onto the sensitive layer, key physical parameters such as its mass, elastic modulus, and electrical conductivity are altered. These changes directly influence the velocity of the surface acoustic wave, which forms the basis of the SAW hydrogen sensor’s response. According to the perturbation theory, and based on the relationship between wave velocity and variations in voltage and phase, the response mechanism of the SAW hydrogen sensor can be expressed as follows [37]:

(1) $$\frac{\Delta v}{v_{0}}=\frac{\Delta f}{f_{0}}=-\frac{\Delta \varphi }{\varphi_{0}}=-c_{m}f_{0}\Delta \rho_{s}+c_{e}f_{0}h\Delta [\frac{4\mu }{{v_{0}}^{2}}(\frac{\lambda +\mu }{\lambda +2\mu })]-\frac{K^{2}}{2}\Delta [\frac{{\sigma_{S}}^{2}}{{\sigma_{S}}^{2}+{v_{0}}^{2}{C_{S}}^{2}}]$$

In this expression: c_m_ and c_e_ are the mass and elasticity sensitivity coefficients, respectively, which characterize the influence of surface mass loading and elastic modulus variations on the acoustic wave velocity; σ_s_ is conductivity of sensitive film; *ρ_s_* (*ρ_s_* = *ρh*) is the mass per unit area of the sensitive film, where *h* is Sensitive film thickness; *λ* and *μ* are the shear modulus and bulk modulus of the film; *K*^2^ is the electromechanical coupling coefficient, reflecting the efficiency of energy conversion between electrical and mechanical energy; C_s_ is the static capacitance per unit length of the piezoelectric crystal. The first term on the right-hand side of Formula 1 represents the mass loading effect, which leads to a negative frequency shift, while the last two terms correspond to the viscoelastic effect and the acoustoelectric effect, respectively. From Formula 1, it is evident that the physical properties of the piezoelectric substrate, along with the density and conductivity of the sensitive film, play a decisive role in the response of SAW sensors. W. Jakubik et al. demonstrated that in SAW sensors with simple metal layer films, the mass loading effect is the dominant response mechanism, whereas in sensors employing metal oxide films, the acoustoelectric effect plays the leading role. The authors tuned the acoustoelectric sensitivity of the sensor by varying the material and thickness of the sensitive membrane. For a single-layer structure, the AE sensitivity reaches its maximum when the acoustic-electric parameter ξ (ξ = σ_s_/v_0_C_s_) equals 0.6. In a bilayer structure, the AE sensitivity is slightly lower than that of a monolayer, and the optimal operating point depends on the relative conductivity ratio x (where x = σ_s1_/σ_s2_) between the first layer (inner layer) and the second layer. For a semiconductor-metal configuration, the metal layer must possess sufficiently high conductivity to achieve maximum AE sensitivity; for a metal-semiconductor configuration, the semiconductor layer must possess sufficiently low conductivity. Furthermore, the maximum AE sensitivity in both single-layer and double-layer structures is strongly influenced by the electromechanical coupling coefficient of the piezoelectric substrate [38,39,40].

Given the decisive influence of the intrinsic properties of piezoelectric materials on sensor performance [41], this paper examines their key physical characteristics. Table 2 summarizes the properties of several commonly used piezoelectric materials, including *v*_0_, *K*^2^, temperature coefficient of frequency (TCF), c_m_, c_e_ and C_s_. Among them, *v*_0_ represents the acoustic phase velocity of the Rayleigh wave propagating along the substrate surface. TCF describes the variation in resonant frequency with temperature, indicating the thermal stability of the substrate. *K*^2^, c_m_, c_e_ and C_s_ have been explained in Formula (1).

These materials are generally classified into two categories: piezoelectric single crystals and piezoelectric films. For piezoelectric single crystals, the type of acoustic wave excitation is determined by the crystal orientation and cutting direction. In gas sensing applications, Rayleigh waves are widely employed due to their high surface sensitivity [42,43], with 128°LiNbO_3_ and Quartz crystals being typical examples. LiNbO_3_ exhibits a high *K*^2^, making it particularly effective for acoustoelectric effects. However, its large TCF renders it susceptible to temperature drift, which is often mitigated by differential detection techniques. Quartz crystals have a large mass sensitivity coefficient, making their mass loading mechanism their primary response mechanism. Moreover, due to their low TCF, quartz crystals are frequently used to minimize response offsets caused by ambient temperature fluctuations [44].

**Table 2:** Piezoelectric material parameters.

Piezoelectric Materials	*v*_0_ (m/s)	*K*^2^ (%)	TCF (ppm/°C)	c_m_ (cm^2^/g/MHz)	c_e_ (cm^2^s/g)	C_S_ (pF/cm)	Ref.
64° Y-X LiNbO_3_	4742	11.3	−70	-	-	-	[45]
128° Y-X LiNbO_3_	3997	5.6	−75	-	-	5.0	[45]
36°Y-X LINbO_3_	4212	4.7	−35	-	-	-	[45]
Y-Z LiNbO_3_	3488	4.8	−94	0.55	1.73 × 10^−7^	4.6	[45,46,47]
Y-Z LiTaO_3_	3230	0.74	-	-	-	-	[47]
ST quartz	3158	0.14	0	1.28	3.86 × 10^−7^	0.55	[45,46]
Y-X quartz	3159.3	0.23	19	1.35	4.16 × 10^−7^	0.55	[45,47]
AIN	5800	1.5–1.7	−19 to −25	-	-	2.12	[48]
ZnO	2720	3.2	−40 to −60	-	-	-	[48]
GaN	4130	0.13	-	-	-	-	[49]

Compared with conventional SAW structures, layered configurations can offer higher sensitivity [50,51]. By epitaxially growing thin piezoelectric films on high-velocity substrates, an effective acoustic waveguide structure can be formed, confining the acoustic wave energy within the piezoelectric layer and thereby enhancing the sensor’s mass-loading sensitivity. Among the available options, ZnO and AIN are the most widely used piezoelectric films in SAW devices. Compared with bulk piezoelectric materials, these films are low-cost, exhibit excellent compatibility with integrated circuit (IC) processes, and can be readily integrated with electronic components for control and signal processing [52]. According to the mass loading mechanism, the higher the operating frequency of the sensor, the greater the frequency shift induced by the mass loading mechanism. The acoustic velocity *v*_0_ is a key parameter in SAW devices design, with its relationship to the resonant frequency *ƒ* and wavelength of the device *λ* is:

(2) $$f=\frac{v_{0}}{\lambda }$$

Equation (2) indicates that, for a constant wavelength, a higher acoustic velocity results in a higher resonant frequency. AIN piezoelectric thin films are widely employed in SAW sensors due to their high acoustic velocity and low intrinsic loss [53,54,55]. However, their broader application is constrained by challenges in thin-film growth processes and their relatively low *K*^2^. Recent studies have shown that *K*^2^ can be improved through optimized deposition techniques and by doping with scandium (Sc) [56]. Compared to AIN films, ZnO films exhibit a higher electromechanical coupling coefficient but a lower phase velocity. Their primary advantage lies in the relative ease of controlling film properties during fabrication. It should also be noted that, in multilayer SAW devices, performance is strongly influenced by dispersion effects, wherein both the phase velocity and electromechanical coupling coefficient vary significantly with film thickness [57]. Finite element modeling (FME) has proven to be an effective tool for analyzing and simulating these effects, and this will be discussed in detail in Section 5.1.

## 4. Design Strategy for SAW Hydrogen Sensors

### 4.1. Hydrogen-Sensitive Film

**Figure 4:**
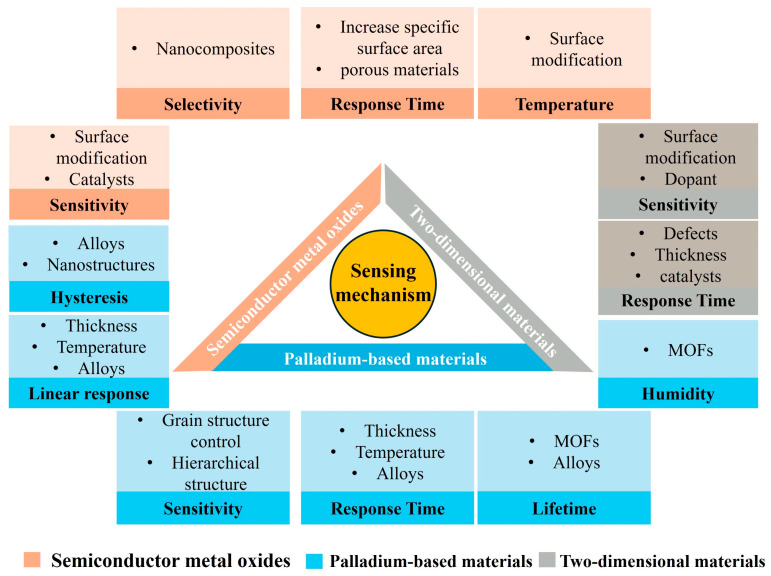
Strategies for improving the performance of SAW hydrogen sensors using different hydrogen-sensitive materials.

Figure 4 summarizes the sensing mechanisms and performance optimization strategies of SAW hydrogen sensors based on three representative hydrogen-sensitive materials: palladium, semiconductor metal oxides (SMOs), and two-dimensional materials. As shown, the intrinsic properties of these materials have a decisive impact on key sensing parameters, including sensitivity, response time, selectivity, linearity, humidity tolerance, and lifetime. To address these challenges and further enhance sensor performance, various strategies—such as doping, surface modification, nanostructure engineering, and the development of composites or alloys—have been extensively investigated. Table 3 summarizes the hydrogen-sensitive films currently employed in SAW sensors along with their corresponding performance metrics. As summarized in Table 3, Palladium-based thin films exhibit superior sensitivity and short response times due to their strong catalytic activity toward hydrogen molecules. In contrast, semiconductor metal oxides such as SnO_2_ and InO_x_ also possess high sensitivity but generally require elevated operating temperatures (150–350 °C), which leads to increased power consumption and prolonged recovery times. This trade-off highlights that while SMO materials are thermally stable and cost-effective, their performance under low-temperature or ambient conditions remains inferior to that of Pd-based films. Therefore, integrating catalytic Pd nanostructures with SMO or two-dimensional materials represents an effective strategy to combine the advantages of both systems, enabling rapid and energy-efficient hydrogen detection.

#### 4.1.1. Palladium-Based Thin Film

Palladium (Pd) is widely recognized as one of the most promising hydrogen-sensing materials due to its high surface activity toward hydrogen molecules. Upon exposure to H_2_, Pd dissociates the molecules into hydrogen atoms, which diffuse into the Pd lattice and gradually form PdH_x_. This process alters the density, Young’s modulus, and electrical conductivity of the Pd film due to electron scattering in PdH_x_ [75]. However, when the H_2_ concentration reaches a threshold of approximately (1–2%), the Pd film undergoes an α-β phase transition, accompanied by structural deformation from lattice expansion and a delayed recovery during hydrogen desorption. As a result, the film cannot fully return to its initial state, leading to a nonlinear response and limiting the sensor’s dynamic range. Various strategies have been proposed to mitigate the α-β phase transition, including the use of Pd nanostructures and Pd alloys. To investigate the influence of hydrogen concentration on the response mechanism of SAW sensors, I. Kerroum et al. analyzed the effects of Young’s modulus and density variations in Pd films at low hydrogen concentrations (0–2%). However, their study did not explicitly identify the dominant contribution to the sensor response [76]. Baile Cui et al. further examined the primary response mechanisms in Pd/Ni films with a 9:1 doping ratio over a wide hydrogen concentration range (100 ppm–38 *v*/*v*%). Their results demonstrated that at hydrogen concentrations below 4 *v*/*v*%, the mass loading effect dominates the sensor response. In contrast, under high hydrogen concentrations, the expansion effect associated with changes in Young’s modulus becomes the key physical parameter leading to response distortion. This distortion can be effectively mitigated by either increasing the operating temperature or reducing the film thickness [77].

**Figure 5:**
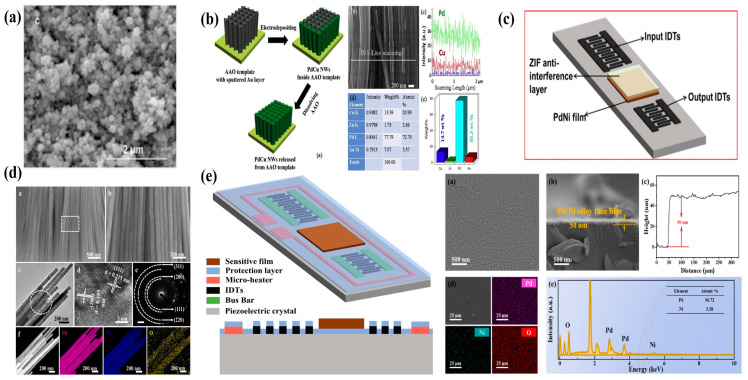
(**a**) SEM image of the surface layer of the palladium nanoporous membrane. (**b**) Synthesis process and characterization of Pd/Cu NWs arrays. (**c**) Characterization of Pd/Ni NWs. (**d**) Morphological and structural characterization of Pd/Ni alloy films. (**e**) Structure of a surface acoustic wave sensor with a micro-heater and characterization of the Pd/Ni structure. (**a**) Reproduced from [58], Copyright (2012), with permission from Elsevier. (**b**) Reproduced from [62], Copyright (2019), with permission from Elsevier. (**c**) Reproduced from [68], Copyright (2023), with permission from Elsevier. (**d**) Reproduced from [61], Copyright (2021), with permission from Elsevier. (**e**) Reproduced from [60], Copyright (2023), with permission from Elsevier.

Pd nanostructures—such as nanowires [61,78], nanotubes [79], nanoparticles [80,81]—offer a high surface-to-volume ratio and nanopore density, providing abundant adsorption sites and accommodating hydrogen-induced morphological changes. This architecture results in faster response and recovery times. For example, in 2013, Constantin Grigoriu’s group fabricated nanoporous Pd nanoparticle films using picosecond pulsed laser ablation, as shown in Figure 5a. The unique porous structure facilitated stress release during expansion, enabling a linear response to hydrogen concentrations of 0.2–0.8% at room temperature, with a sensitivity of 0.31 Hz/ppm and a detection limit of 48 ppm. However, the response time remained relatively long at 15–44 s [58]. In 2014, I. Rýger et al. demonstrated an alternative approach by employing a GaN/SiC heterostructure to confine acoustic energy within an epitaxial waveguide. A 100 nm Pd sensing layer achieved a maximum frequency shift of 60 kHz at 1000 ppm H_2_ and a response time of 15 s. Nevertheless, the recovery time was still prolonged (about 5 min) due to inherent Pd film limitations [51].

Another promising strategy to accelerate recovery involves doping Pd membranes with noble metals, which induces pre-expansion of the lattice before hydrogen exposure. Currently, various precious metal-doped Pd alloys have been extensively investigated [23,82,83]. Meddy Vanotti et al. compared the effects of pure Pd and Pd–Y alloys on sensor response. Compared with pure Pd, Pd–Y exhibits higher physical and mechanical strength and can effectively suppress the α–β phase transition of the Pd film, resulting in an approximately 1.7-fold increase in phase shift. However, the sensor response still shows a nonlinear relationship, which may be caused by dispersion effects due to the guidance of elastic waves [69]. Wang Wen and his research team fabricated Pd/Cu nanowires on anodized aluminum oxide (AAO) templates via electroplating, as shown in Figure 5b. Alternating doping with Cu atoms effectively suppressed the α–β phase transition of PdH_x_. The resulting sensor demonstrated a highly linear response over the 0.1–4.5% dynamic range, with a sensitivity of 1.5 kHz/%, and both response and recovery times below 4 s at room temperature [62]. To evaluate the contribution of different mechanisms, Al thin films were deposited on the acoustic wave transmission area to eliminate the acoustoelectric coupling effect. Sensors with Al coatings exhibited almost no measurable response, confirming that the acoustoelectric effect is the dominant sensing mechanism. Pd/Ni alloys with various doping concentrations have also been studied extensively [84,85]. Xinyu Wang et al. synthesized uniform Pd/Ni nanowires (NWs) using anodized aluminum templates, as shown in Figure 5d, achieving a sensitivity of 1.65 mV/% for 0.3–3.5 *v*/*v*% H_2_, with response/recovery times of less than 2 s/4 s. The study indicated that thicker Pd films yield higher sensitivity but at the cost of longer response/recovery times and greater acoustic attenuation [61]. Notably, most Pd-based hydrogen sensors are tested in nitrogen environments, neglecting the adsorption effects of H_2_O, CO, and SO_2_ on active sites, which can reduce hydrogen uptake. Potential solutions include introducing an anti-interference layer or performing surface heat treatment [86,87]. For example, the research groups led by Baile Cui [60] and Qi Liu [88] investigated the effect of operating temperature on Pd/Ni thin films (with a doping ratio of 96:4), with device structure and thin film characterization shown in Figure 5e. The study demonstrated that at an operating temperature of 75 °C, adsorption and desorption processes were highly consistent, response/recovery times were significantly reduced, nonlinear error decreased, and long-term stability improved. As shown in Figure 5c, Jing Jin et al. introduced ZIF-8, a highly porous conductive metal–organic framework, as an anti-interference layer. Through molecular sieving, ZIF-8 enhanced H_2_ selectivity and mitigated interference from other gases [68]. Overall, the development of Pd alloys and nanostructures has markedly improved response times and expanded the dynamic range of SAW hydrogen sensors. However, systematic studies on the effect of different doping levels on the sensing mechanism remain limited. Further research should focus on optimizing film microstructure and grain size to further enhance performance [89,90].

#### 4.1.2. Semiconductor Metal Oxides

**Figure 6:**
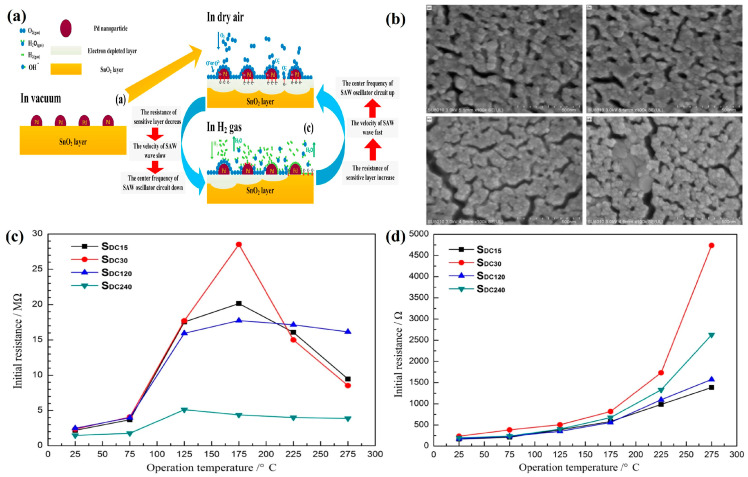
(**a**) Sensing mechanism based on Pd-SnO_2_ sensitive film. (**b**) FE-SEM images of Pd films with different thicknesses on SnO_2_ films. (**c**) Initial resistance of SnO_2_ films with different thicknesses of Pd surface modification in dry air at different temperatures. (**d**) Initial resistance of SnO_2_ films with different thicknesses of Pd surface modification in dry nitrogen at different temperatures. Reprinted from [64], copyright (2016), with permission from Elsevier.

Compared with the long-term stability and poisoning issues of Pd thin films in air, SMO-based hydrogen sensors are widely used due to their lower cost, excellent thermal stability, and chemical resistance. Among them, n-type semiconductors—such as SnO_2_, ZnO, and WO_3_—are the most commonly employed hydrogen-sensing materials. The sensing mechanism can be explained as follows: O_2_ molecules adsorb onto the material surface and capture electrons from its conduction band. Depending on the ambient temperature, various ionized oxygen species are formed (O_2_^−^ at temperatures below 100 °C, above 100 °C as O_−_ and O_2−_), which increases the depletion layer and the initial resistance of the SMO. When hydrogen gas is present, it reacts with these ionized oxygen species via a reduction reaction, releasing electrons back to return to the SMO conduction band and thereby enhancing its conductivity (Figure 6a illustrates the sensing mechanism of the sensor exposed to Pd-SnO_2_). To enhance the contribution of the piezoelectric effect to the overall response, most SMO-based SAW hydrogen sensors currently use LiNbO_3_ as the piezoelectric substrate due to its large piezoelectric coupling coefficient.

Additionally, because pure SMO exhibits limited selectivity, various strategies—such as surface modification, metal doping, and heterostructures—have been investigated to improve sensitivity and selectivity. For pure SMO, employing Pd or Pt catalysts to activate gas molecules is a mainstream approach, as these precious metals lower the activation energy required for gas adsorption and desorption. For example, Wang Cheng et al. prepared InO_x_ layers using radio frequency diode sputtering and used Pt as a catalyst. At room temperature, the sensor exhibited a frequency shift of 11.83 kHz for 400 ppm of hydrogen [66]. Liu Yang et al. [64] used direct current magnetron sputtering to prepare SnO_2_ thin films with Pd surface modification as the sensitive film for the sensor, as shown in Figure 6b. With increasing Pd film thickness, the Pd film developed a highly dispersed and discontinuous morphology. As shown in Figure 6c,d, the initial resistance of the film was measured in dry air and dry nitrogen. The study showed that the initial resistance of the film was mainly affected by the oxygen content in the air. At the optimal operating temperature of 175 °C, the sensor achieved a maximum frequency shift of 115.9 kHz at a hydrogen concentration of 2000 ppm, with response/recovery times of 1 s and 583 s, respectively. The prolonged recovery time was likely due to water molecules generated during the chemical reaction blocking the adsorption of oxygen atoms. Furthermore, as shown in Figure 6d, thicker Pd films and high temperatures can cause excessively low initial resistance, thereby degrading sensor performance. SMO sensors based on various surface modifications have shown promising application prospects. However, further research is required to lower operating temperatures, enhance selectivity, and reduce humidity interference. Owing to the spillover effect of precious metal catalysts, catalyst particles must be uniformly dispersed over the SMO surface to effectively regulate hydrogen sensing. Future studies should focus on developing nanoporous materials with tunable pore diameters and high porosity to ensure uniform catalyst particle distribution.

**Figure 7:**
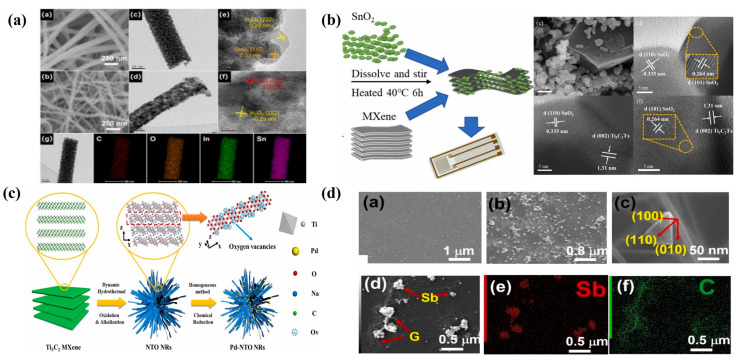
(**a**) Morphological characterization of ln_2_O_3_-SnO_2_ NPs. (**b**) Preparation scheme of Pd-NTO NRs. (**c**) MXenes-SnO_2_ heterostructure and electron mapping image; (**d**) TEM image of Sb/G heterostructure. (**a**) Reproduced from [91], Copyright (2024), with permission from Elsevier. (**b**) Reproduced from [94], Copyright (2023), with permission from Elsevier. (**c**) Reproduced from [95], Copyright (2022), with permission from Elsevier. (**d**) Reproduced from [96], Copyright (2023), with permission from Elsevier.

Given that SMO hydrogen-sensing materials have been extensively investigated in the field of resistive sensing, this paper presents recent advances in resistive hydrogen-sensing materials and provides a theoretical basis for the design of SAW hydrogen-sensing materials. Qiaoling Xing [91] introduced ln ions to induce lattice distortion in SnO_2_ crystals, thereby increasing the number of oxygen defects in ln_2_O_3_-SnO_2_ nanoparticles (NPs) and significantly enhancing hydrogen-sensing performance, as shown in Figure 7a. The prepared ln_2_O_3_-SnO_2_ nanofibers (NFs) exhibited excellent structural uniformity, with an average pore size of 8.1 nm and a specific surface area of 92.9 m^2^/g. This high specific surface area and small pore size facilitate rapid diffusion and adsorption of gas molecules, greatly improving the response speed. The difference in work function between the n-n type heterostructure results in a redistribution of the Fermi level at the interface, forming an electron depletion layer and an accumulation layer. This band-modulation effect enhances the internal electric field at the interface, thereby accelerating oxygen ionization. Studies have shown that the sensor achieves optimal response at 350 °C. In recent years, hydrogen-sensing materials based on SMOs have evolved from traditional single/double-layer films to advanced two-dimensional (2D) layered nanocomposites [92,93].

#### 4.1.3. Other Hydrogen-Sensitive Materials

Compared with one-dimensional materials, 2D materials with atomic-layer thickness provide new opportunities for constructing high-performance hydrogen sensors, owing to their abundant surface-active sites and tunable electronic structures. Their sensing mechanism is based on variations in electrical conductivity upon hydrogen adsorption, enabling the detection of hydrogen concentration. Among them, MXenes and graphene have demonstrated considerable potential in hydrogen energy systems. MXenes constitute a novel class of materials synthesized by etching layered transition metal carbides, nitrides, and carbonitrides, with a structural formula represented as M_n+1_X_n_T_x_ (for a detailed discussion of MXenes materials, please refer to [97]). Heterostructures composed of MXenes and SMOs exhibit higher hydrogen adsorption energy compared with pure SMO structures. Qingdong Chen et al. [94] combined high-temperature-calcined SnO_2_ with multilayer MXenes to fabricate MXenes-SnO_2_ composite films (as shown in Figure 7b). Compared with pure SnO_2_, the composite sensor exhibited significantly enhanced responsiveness to hydrogen gas. The MXenes-SnO_2_ sensor with a doping mass fraction of 10 wt% achieved the highest response, with a response time of 11 s and a minimum detection limit of 1.81 ppm. As shown in Figure 7c), Junbiao Wu et al. [95] employed a dynamic hydrothermal method using Ti_3_C_2_ MXenes as the precursor to synthesize sodium titanate nanoribbons (NTO NRs) rich in edge-site oxygen vacancies. When modified with Pd nanoparticles, the sensor achieved a response time of 1.1 s at room temperature for 1% hydrogen, with a detection range of 0.8 ppm to 10%. As described in Figure 7d, Neeraj Kumar et al. proposed a 2D Sb/graphene heterostructure hydrogen sensor, which exhibited an exceptionally low detection limit of 50 ppb and excellent long-term stability. This superior performance was attributed to the abundant active sites created by the triangular morphology of Sb and the tunable Schottky barrier in the Sb/G heterostructure [96]. Sorin Vizireanu et al. employed vertically aligned graphene layers as the sensing material in a SAW sensor. By applying plasma treatment, the average sensitivity of the sensor was enhanced to 9.4 Hz/ppm, while the detection limit was reduced from 13.35 ppm to 7.79 ppm [72].

Overall, unlike traditional SMO hydrogen sensors that require high-temperature activation, 2D materials can generate abundant active sites through the integration of dopants, nanoparticles, and other materials to form heterointerfaces. Furthermore, their adjustable interlayer spacing facilitates efficient gas adsorption and carrier migration at room temperature, making them promising candidates for the development of low-power hydrogen sensors.

### 4.2. Design of Interdigital Transducer

#### 4.2.1. Material Selection and Interdigital Transducer Structure

As the excitation component of SAW devices, the interdigital transducer (IDT) plays a critical role in determining the acoustic–electric conversion efficiency, resistive losses, and long-term reliability of the device. The selection of electrode materials directly influences these parameters. Low-resistance materials are generally preferred to minimize series resistance during signal transmission. In addition, the acoustic impedance of the electrode material should be well-matched with that of the piezoelectric substrate to reduce acoustic wave reflection and scattering losses at the interface. Thermal stability is also an essential consideration. 

Table 4 summarizes the key physical parameters of several common IDT materials. Mo and Au are often employed in high-temperature SAW sensors due to their excellent resistance temperature characteristics and thermal stability [54,98,99]. However, because of their poor adhesion to the piezoelectric substrate (“adhesion” refers to the interfacial adhesion performance between the electrode material and a common piezoelectric substrate such as LiNbO_3_, LiTaO_3_, or quartz crystal), a thin Ti layer is often deposited beneath them as an adhesion layer to enhance interface stability. Al has low acoustic impedance and low density, which enables effective propagation of acoustic signals [100], but it softens at elevated temperatures and is therefore mainly used in SAW sensors operating at low to moderate temperatures. Moreover, Al is susceptible to corrosion and has relatively poor mechanical strength; to address these issues, a SnO_2_ passivation layer is commonly applied as a protective coating.

#### 4.2.2. Configuration of Interdigital Transducer

**Figure 8:**
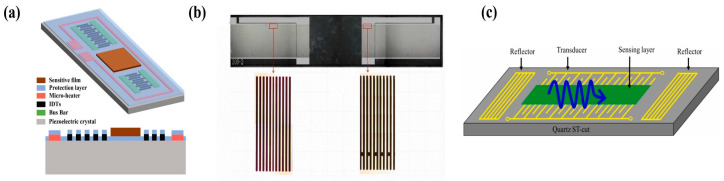
(**a**) Delay line configuration. (**b**) Two-terminal resonator configuration. (**c**) Single-port resonator configuration. (**b**) Reproduced from [101], Copyright (2023), with permission from Elsevier. (**c**) Reproduced from [102], Copyright (2023), with permission from Elsevier.

As shown in Figure 8, according to the layout of the interdigitated electrodes, SAW sensors can be classified into delay line type (As shown in Figure 8a) and resonator type (as shown in Figure 8b,c). For the delay line type, the acoustic wave signal is introduced through the input IDT, propagates along the substrate surface, and is received by the output IDT on the other side. Its primary advantage lies in providing a larger sensing area, which facilitates the deposition of the sensitive film and enhances gas-sensitive response. Resonator-type sensors are further divided into single-port resonators and dual-port resonators. These devices incorporate periodic reflective grating structures on both sides of the IDT to reflect propagating acoustic waves, forming standing wave fields forming standing wave fields and producing strong resonance at specific operating frequencies. The design focus of this structure is to enhance the device’s quality factor (Q-value) to achieve high sensitivity and high resolution, making it suitable for high-precision detection applications.

**Figure 9:**
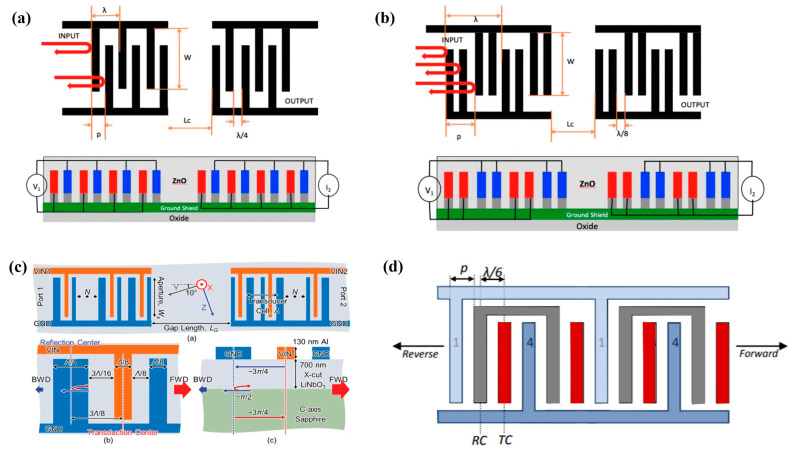
(**a**) Uniform Electrode Configuration. (**b**) Split-finger Electrode Configuration. (**c**) Single-phase Unidirectional Transducer Electrode Configuration. (**d**) Floating Electrode Unidirectional Transducers Configuration. (**a**,**b**) Reproduced from [103], Copyright (2013), with permission from IEEE. (**c**) Reproduced from [104], Copyright (2021), with permission from CC BY 4.0. (**d**) Reproduced from [105,106], Copyright (2019), with permission from Pleiades Publishing.

Unlike traditional SAW filter designs, SAW sensors are not primarily concerned with the shape of the transmission curve. Instead, their design emphasizes low insertion loss and linear phase characteristics within the passband. The main source of sensor nonlinearity stems from the superposition of IDT reflection signals and main signals. Therefore, how to suppress IDT acoustic reflections has become a critical challenge in SAW sensor development. Currently, several common IDT configurations have been developed, as shown in Figure 9, including uniform interdigitated transducers, split-finger transducers, single-phase unidirectional transducers (SPUDTs), and floating electrode unidirectional transducers (FEUDTs). Among these, the uniform interdigitated transducer is the simplest to fabricate, with electrode widths and spacings both set to λ/4. However, due to the use of a single-electrode configuration, acoustic waves propagate in both directions, leading to a theoretical inherent insertion loss of 6 dB. The split-finger structure modifies the single-electrode configuration by dividing it into multiple narrower electrode fingers, producing an approximate 180° phase difference between adjacent electrodes. This arrangement effectively cancels higher-order harmonic signals, thereby reducing nonlinear responses [49,103]. The SPUDT design introduces an asymmetric layout, consisting of a pair of conducting electrodes and a reflective electrode [104], enabling unidirectional propagation of acoustic waves and reducing insertion loss. The unidirectionality behavior can be explained by the phase path: the forward wave generated at the conducting center experiences a phase delay of −3π/4 when it reaches the reflective center. After reflection, a π/2 difference is introduced, and upon returning to the conducting center, it undergoes another phase delay of −3π/4. The phase difference of −2π results in constructive interference with the forward wave, while the backward wave undergoes destructive interference. Building on this concept, FEUDTs incorporate floating electrodes, further enhancing unidirectional transmission by adjusting electrode width and position [105,106]. In addition to electrode configuration, parameters such as the IDT number, acoustic aperture, and delay-line distance must be comprehensively optimized. Increasing the number of IDT pairs can reduce insertion loss and return loss, but also affects the device’s relative bandwidth. To suppress excess mode interference, the left transducer should be as long as possible to ensure the relative bandwidth remains within a narrow range [107]. The number of IDTs also influences port impedance, which must be matched to the external measurement system to minimize signal reflections [54,108]. A larger acoustic aperture can effectively suppress edge diffraction, thereby reducing passband ripple; however, excessively long apertures increase the risk of short-circuiting, so typical designs select values within the range of 50λ–200λ. Similarly, the delay-line distance should be optimized to prevent insertion loss and in-band ripple caused by diffraction. Given the intrinsic relationship between wavelength and frequency, selecting an appropriate IDT configuration requires balancing sensitivity with fabrication stability [109].

## 5. Simulation and Modeling of SAW Device

To simulate and design SAW devices, the use of reliable equivalent models is essential. Several modeling approaches are currently employed, including the finite element method (FEM), equivalent circuit models, and coupling-of-modes (COM theory) [107].

### 5.1. FEM Method

**Figure 10:**
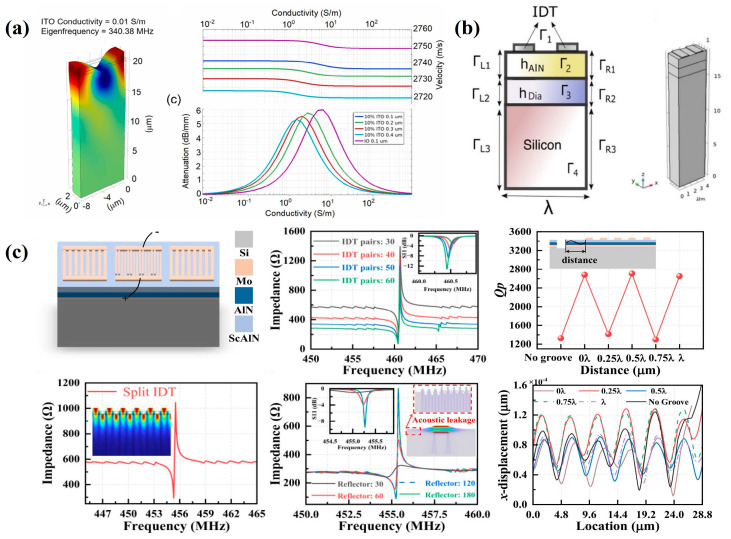
(**a**) Simulation of indium tin oxide/langasite SAW resonator. (**b**) Schematic of the AlN/diamond/Si SAW device structure. (**c**) Simulation of a single-port AlN/ScAlN composite thin film SAW device. (**a**) Reproduced from [70], Copyright (2021), with permission from Elsevier. (**b**) Reproduced from [114], Copyright (2024), with permission from The Minerals, Metals & Materials Society. (**c**) Reproduced from [54], Copyright (2024), with permission from Elsevier.

FEM provides a powerful numerical simulation technique for the propagation of surface acoustic waves in piezoelectric materials, as defined by their governing differential equations [110,111]. COMSOL Multiphysics 6.0 provides an effective simulation platform for optimizing SAW device structural parameters through multiphysics coupling models. Since IDTs are periodically arranged, most simulation studies adopt a two-dimensional single-period model to reduce computational degrees of freedom. The following is a report by researchers on FEM analysis of SAW devices, as shown in Figure 10a, Jagannath Devkota et al. developed a two-dimensional equivalent model of a reflective delay line structure to predict the operating frequency and evaluate the influence of a tin-doped indium oxide sensing layer on device performance. Their analysis examined how sensing layer conductivity affects resonance frequency, wave velocity, and the effective electromechanical coupling coefficient, demonstrating that conductivity can be tuned by controlling tin doping levels to optimize hydrogen sensor sensitivity [70]. Massood Z. Atashbar et al. applied FEM modeling to a SAW hydrogen sensor with a delay-line configuration. When the Pd film was exposed to a hydrogen environment below 3%, the output voltage exhibited a time delay of 1.4 ns [112]. As shown in Figure 10b, Ziad Abu Waar, S. Maouhoub have conducted FEM analysis on layered SAW devices, revealing that device performance is closely related to electrode height, normalized film thickness, and mass loading [113,114]. As shown in Figure 10c, Yuanhang Qu et al. established a two-dimensional model of a single-port SAW resonator to investigate the effects of IDTs and reflective gratings on impedance response in the frequency domain [54]. Additionally, Nimmala Harathi et al. reported a SAW device employing ZnO as the sensing layer and LiNbO_3_ as the piezoelectric substrate, using FEM to analyze its operating frequency under gaseous and non-gaseous conditions [115]. These studies demonstrate that the FEM method has become an indispensable tool for the structural analysis and performance optimization of SAW devices.

### 5.2. Equivalent Circuit Model

**Figure 11:**
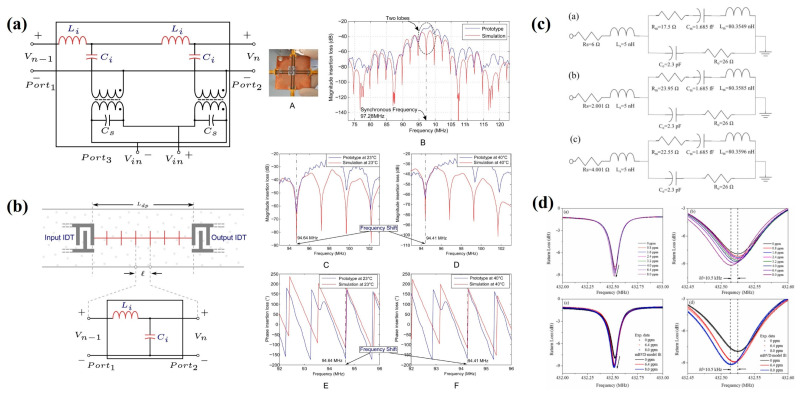
(**a**) Single-cycle IDT model. (**b**) Delay Path Model. (**c**) Modified MBVD equivalent circuit model for 0 ppm, 6.4 ppm, 8.0 ppm. (**d**) Comparison of experimental data and MBVD model. (**a**,**b**) Reproduced from [119], Copyright (2016), with permission from Elsevier. (**c**,**d**) Reproduced from [102], Copyright (2023), with permission from Elsevier.

Equivalent circuit models are widely applied in circuit analysis to represent the complex acoustic–electrical coupling mechanisms and acoustic wave propagation in SAW devices as a set of quantifiable lumped or distributed circuit elements [116]. This abstraction enables precise characterization of device electrical performance, facilitates integration into various commercial simulation platforms, and supports impedance matching with peripheral circuits as well as accurate prediction of insertion loss and frequency response [117,118]. Consequently, equivalent circuit models serve as an important reference for readout circuit design. L. Perez-Cortes et al. proposed an enhanced SAW electrical model, as shown in Figure 11a,b. This model extends the Mason equivalent circuit model by replacing the original T-type impedance network with inductance (L) and capacitance (C) components. Validation against experimental data from prototype SAW devices under varying temperatures demonstrated a high degree of agreement, confirming the model’s predictive accuracy [119]. As shown in Figure 11c,d, Jitendra Singh employed the modified Butterworth–Van Dyke (MBVD) equivalent circuit model to simulate changes in the electrical behavior of a sensor when exposed to a target gas. In this model, the equivalent parameters are obtained through vector network analyzer measurements and fitted from the data. The structure comprises a parallel configuration of a motional branch and a static branch, where the motional resistance (R_m_) and motional inductance (L_m_) are influenced by gas concentration [102]. Overall, the equivalent circuit model provides valuable insight into the electrical response of SAW sensor chips under external environmental disturbances, offering a critical tool for comprehensive circuit-level analysis and optimization.

## 6. Fabrication Method of SAW Devices

Typical SAW devices are usually fabricated using photolithography, with the patterning of the IDT region guided by this process. The IDT line widths in SAW devices are generally in the micrometer or sub-micrometer range. For different etching techniques, wet etching is suitable for structures with line widths larger than 4 µm, offering high yield but causing slight line width loss. Lift-off processes are suitable for line widths of 2–4 µm, which may slightly broaden the metal lines. Dry etching is typically used for line widths of 1–2 µm and requires high precision to avoid device shorting. Electron beam lithography is generally employed for sub-micrometer devices.

**Figure 12:**
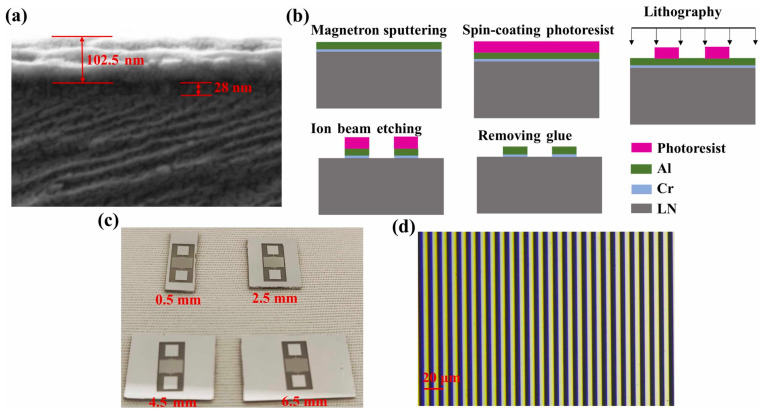
(**a**) The SEM image of the electrode cross-section. (**b**) Fabrication processes for SAW sensors. (**c**) The physical drawings of SAW sensors. (**d**) The optical image of the electrode surface. Reproduced from [121], Copyright (2025), with permission from Elsevier.

The typical manufacturing process is shown in Figure 12. The fabrication process of SAW devices begins with cleaning the piezoelectric substrate to remove surface residues. Subsequently, the required metal thin film is deposited using techniques such as electron beam evaporation or magnetron sputtering, and the film thickness can be controlled by adjusting the deposition rate. The wafer is then placed on a hot plate to remove surface moisture and enhance adhesion of the photoresist, followed by spin-coating of the photoresist, with thickness controlled via spin speed and acceleration. After a soft bake to harden the photoresist, the wafer is exposed through a mask, typically using contact or proximity exposure to ensure high resolution and minimize defects [120]. The exposed wafer is then developed in a compatible developer to remove unexposed or unwanted photoresist areas, completing the pattern transfer. Finally, the metal IDT pattern is formed using wet etching, dry etching, or lift-off processes.

## 7. Read Circuit

The design of SAW devices should be approached as an integrated system comprising two core components: the SAW chip and the signal readout circuitry. Key parameters—including the IDT configuration, delay line length, and hydrogen-sensitive thin-film properties—jointly affect the sensor’s insertion loss and linearity [122,123]. The readout circuit must not only convert the hydrogen concentration signal into a measurable electrical output, but also be designed in accordance with the underlying acoustic mechanism of the device. Specifically, its port impedance, gain, and bandwidth should be properly matched to the device in order to minimize noise and avoid interference arising from excessive gain. Accordingly, signal processing strategies should be closely integrated with the sensor’s structural design to achieve optimal sensing performance. Based on their operating principles, existing readout circuits for SAW hydrogen sensors can generally be classified into two main categories: differential oscillation circuits and phase detection circuits.

### 7.1. Differential Oscillation Circuit

**Figure 13:**
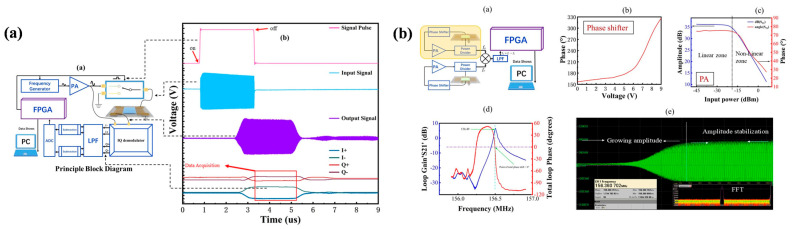
(**a**) Phase detection circuit and output signal schematic diagram. (**b**) Oscillation circuit and output signal schematic diagram. (**a**,**b**) Reproduced from [124], Copyright (2025), with permission from Elsevier.

As shown in Figure 13b, a classic feedback oscillation circuit generally consists of a transistor matching circuit, a feedback network, a phase shifter, an output filter, and a SAW sensor [124]. To satisfy the oscillation conditions, the amplifier must provide a gain greater than the total loop loss to sustain oscillation. In the feedback branch, the SAW sensor functions analogously to an LC resonant circuit, serving as a frequency-selective element to enable self-oscillation. The phase shifter ensures that the total phase shift of the loop is an integer multiple of 0 or 2π, while the output filter suppresses low-frequency interference signals generated within the circuit. The frequency stability of the oscillator has a direct impact on the sensor’s gas detection limit. For instance, the differential oscillator developed by Wen Wang et al. achieved short-term and medium-term frequency stabilities of ±1.5 Hz/s and ±20 Hz/h, respectively, at room temperature [62]. However, this performance is constrained by the need for the amplifier to operate at high gain, which in turn degrades frequency stability. In the case of delay-line-type SAW sensors, longer sensitive film coating regions are often employed to enhance sensitivity by increasing the gas–sensor interaction area. Nevertheless, this approach typically introduces higher insertion loss, which can lead to oscillator startup failure [61], thereby limiting its applicability in such configurations.

### 7.2. Phase Detection Circuit

The operating principle of the phase detection circuit involves generating RF signals at a specified frequency on the input IDT, then comparing their phase with the output signal to extract the phase difference as the sensing parameter. Unlike oscillation circuits, phase detection circuits do not depend on high amplifier gain. Instead, the key to detection lies in the sensor’s linear phase response. For instance, the phase-matching circuit developed by Xinyu Wang et al. demonstrated a low baseline noise of only 0.45 mV [61]. Chen Fu conducted a comparative study of SAW sensors fabricated on ST-quartz and LiNbO_3_ substrates. On the ST-quartz substrate, the split-finger electrode structure improved phase linearity; however, it did not significantly enhance the phase linearity of LiNbO_3_, which possesses a large electromechanical coupling coefficient. This limitation is mainly attributed to IDT reflections influenced by mass loading and acoustoelectric coupling effects, as shown in Figure 13a. The authors further demonstrated that sensor linearity could be improved from 0.916 to 0.995 by controlling the excitation signal duration and the output signal time window to achieve temporal separation between the main signal and the triple transit response [125].

## 8. Challenges Faced by SAW Hydrogen Sensors

**Figure 14:**
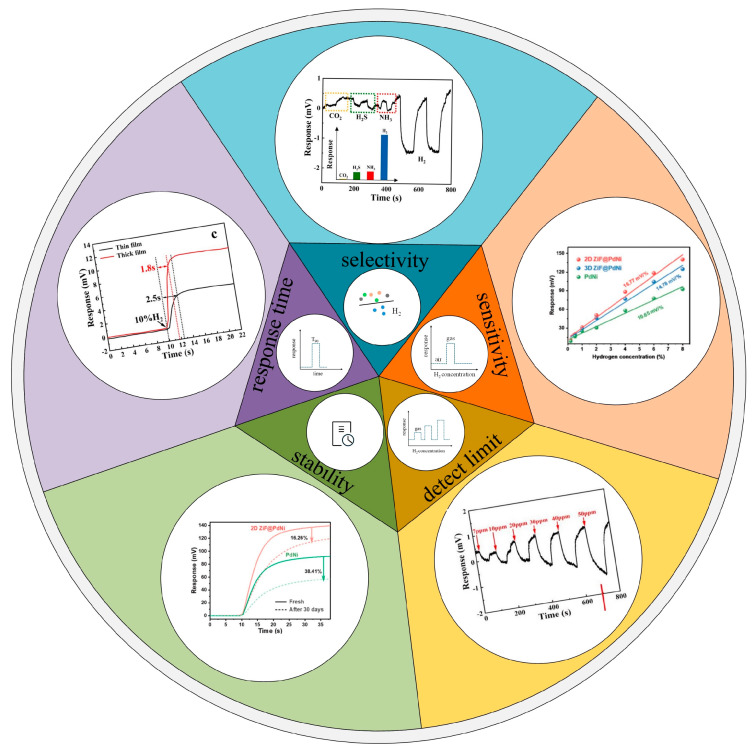
Representative performance indicators of SAW hydrogen sensors, including selectivity, sensitivity, response time, stability, and detection limit.

The development of SAW technology has demonstrated great potential for hydrogen-sensing applications. However, several challenges remain before this technology can transition from experimental setups to commercially viable devices. These challenges are primarily associated with critical performance parameters, including sensitivity, selectivity, response time, detection limit, and long-term stability, as shown in Figure 14.

These aspects are discussed in detail below:

(1) Long-term stability and selectivity: While SAW sensors themselves exhibit excellent stability, the failure mechanisms of hydrogen-sensitive materials during operation require further investigation. Material degradation, oxidation, and poisoning are the primary factors leading to response deterioration. In addition, various types of cross-sensitivity remain unresolved. Recent studies have shown that the molecular sieving properties of MOF or polymer coatings offer an effective strategy to enhance the long-term stability of hydrogen-sensitive materials.
(2) Response/recovery time: Material innovation plays a decisive role in significantly shortening the response and recovery times of SAW hydrogen sensors. The introduction of nanostructures, alloying strategies, and catalytic modifications can substantially increase the specific surface area and the number of active adsorption sites for hydrogen molecules, thereby accelerating reaction kinetics. Additionally, effective surface treatments and structural-level wavelength optimization have been shown to further improve response performance. Recent reports indicate that SAW hydrogen sensors can achieve response times of less than 2 s. However, this performance still falls short of the stringent requirements specified by international standards such as ISO for certain applications. Furthermore, as most hydrogen sensors are currently tested in nitrogen atmospheres, further studies are needed to develop high-performance sensors capable of reliable operation in ambient air.
(3) Sensitivity: According to the response mechanism of SAW devices, the sensitivity of a sensor primarily depends on the structural parameters and thickness of the sensitive film, as well as the device’s operating frequency. However, current SAW hydrogen sensors still fall short of achieving ppb-level sensitivity, largely because their operating frequencies are limited to the MHz range. Although epitaxial waveguides have been reported to confine acoustic energy on the surface of the piezoelectric layer and thereby improve sensitivity, their practical application remains constrained by fabrication complexity and the coupling of multiple acoustic modes. Consequently, future research should focus on elucidating the influence of thin-film structures on the response mechanism of SAW devices and exploring the feasibility of GHz-level SAW architectures, with the goal of achieving breakthroughs in sensitivity and detection limits to ultimately approach ppb-level performance.
(4) Interference resistance: To enhance the sensor’s performance in high-temperature and high-humidity environments, appropriate compensation measures are essential. Temperature compensation is mainly achieved through two methods: material selection—such as using ST-X quartz substrates with a near-zero TCF—and structural design, like differential detection. In humid conditions, competitive adsorption of water molecules can compromise baseline stability. Future research could focus on innovative packaging materials or real-time algorithmic compensation within the signal processing framework [126].
(5) Limit of Detection (LOD): The LOD of a sensor is typically evaluated based on the system’s baseline noise and the sensor’s sensitivity. Achieving breakthroughs in LOD requires simultaneously reducing system noise and enhancing sensitivity, which depends not only on the design of high-performance SAW chips but also on their integration with low-noise readout circuits. Compared with conventional differential oscillator circuits, phase-detection circuits can circumvent the noise amplification caused by oscillator gain limitations, thereby enabling lower baseline noise and higher resolution. This approach provides a feasible pathway for SAW hydrogen sensors to achieve ppb-level detection limits.

## 9. Conclusions

In summary, this review systematically analyzes the response mechanisms of SAW hydrogen sensors and explores performance optimization strategies from three core aspects: sensing materials, device structures, and signal processing. The preceding sections revealed that the sensing performance of SAW devices is essentially determined by the interplay between hydrogen-sensitive films and piezoelectric substrates. Palladium-based thin films, semiconductor metal oxides, and emerging two-dimensional materials show significant potential. Through nanostructure engineering, alloying modifications, and catalytic treatment, the sensitivity and response speed of these materials have been effectively enhanced. Nevertheless, they still face challenges in long-term stability and interference resistance, particularly in complex ambient air where adsorption effects and cross-sensitivity remain critical issues. In device design, the construction of layered structures and the optimization of interdigital transducers (IDTs) can effectively confine acoustiv wave energy, thereby improving the sensor’s sensitivity and linear response. Meanwhile, simulation tools such as finite element analysis (FEM) provide essential theoretical support for parameter optimization and performance prediction. Regarding signal processing, oscillation circuits and phase-detection circuits are two mainstream readout schemes. Oscillation circuits are simple in structure and convenient to use, but their frequency stability is limited by amplifier gain. In contrast, phase-detection circuits can overcome this limitation, effectively reduce baseline noise, and provide a feasible pathway to achieve lower detection limits.

Future research may further focus on multifunctional sensing films, high-frequency device architectures, and optimized signal processing strategies to surpass ppb-level detection thresholds and achieve long-term stable operation. Through coordinated optimization of materials, structures, and circuits, SAW hydrogen sensors are expected to evolve into miniaturized, reliable, and commercially viable solutions for hydrogen safety monitoring.

## Figures and Tables

**Table 1:** ISO hydrogen sensor performance requirements and the service life of current commercial sensors [10].

Project	Detection Range	LOD	Response (t90)/Recovery Time (t10)	Accuracy	Ambient Temperature (°C)	Ambient Humidity (RH%)	Ambient Pressure (kPa)
Stationary	0.01–10 vol%	<0.01–0.05 vol%	<30 s/60 s	±10%F.S.	−20–60	15–95	80–120
Automobile	0.01–10 vol%	<0.01–0.2 vol%H_2_	<1–3 s/30 s	±5–10%F.S.	−40–105	5–95	80–120

**Table 3:** Summarizes the hydrogen-sensitive films currently employed in SAW sensors along with their corresponding performance metrics.

Sensitive Film	Range	LOD	Sensitivity	Response Time	Recovery Time	Operating Temperature	Ref.
Nanoporous Pd	0.008–2%	48 ppm	0.31 Hz/ppm	15–44 s	34 s	-	[58]
Pd-CuP_C_	0.5–4%	-	0.1048 Hz/ppm	-	-	38 °C	[59]
Pd/Ni	0.2–10%	15 ppm	0.181 mv/%	<2 s	<7 s	75 °C	[60]
Pd/Ni NWs	0.3–3.5%	7 ppm	1.65 mv/%	<2 s	<4 s	RT	[61]
Pd/Cu NWs	0.1–4.5%	7 ppm	1.5 kHz/%	<4 s	<4 s	RT	[62]
Pd/Ni NWs	1–3%	-	0.92 mv/%	<2 s		RT	[63]
Pd-SnO_2_	0.01–0.2%	-	115.9 kHz to 2000 ppm	1 s	583 s	175 °C	[64]
Pd (GaN/SiC)	0.002–0.1%	20 ppm	60 kHz to 1000 ppm	15 s	5 min	RT	[51,65]
Pt/InO_x_	0.04–0.68%	-	23.63 kHz to 2000 ppm	-	-	RT	[66]
Polyaniline/WO_3_	1–4%	-	-	-	-	-	[67]
PdNi/ZIF-8	0.2–8%	5 ppm	16.77 mv/%	-	-	RT	[68]
Pd-Y	0.5–2%	-	-	-	-	-	[69]
ITO	5–100%	-	0.0018 rad/vol%	-	-	350 °C	[70]
ZnO NWs	0.2–2%	2253 ppm	0.062 Hz/ppm	-	-	RT	[71]
Carbon Nanowalls	0.02–0.1%	7.79 ppm	9.4 Hz/ppm	-	-	RT	[72]
Pd/ZnO	0.2–2%	59 ppm	0.51 Hz/ppm	12 s–16 s	-	RT	[73]
Pd/Gr	0.25–1%	-	30 kHz to 1%	-	-	RT	[74]

**Table 4:** Properties of different electrode materials.

Performance Parameters	Al	Au	Cu	Mo	W
Adhesion	Robust	Poor	Robust	Robust	Good
Density (g/cm^3^)	2.7	19.32	8.96	10.28	19.25
Maximum operating temperature (°C)	250	300	250	500	600
Resistivity (μΩ/cm)	2.65	2.2	1.7	5.34	5.0

## Data Availability

No new data were created or analyzed in this study.
